# Geranylgeraniol as a Modulator of Mevalonate Pathway Disruption: A Scoping Review of Cellular Mechanisms and Skeletal Outcomes in Osteoporosis Models

**DOI:** 10.3390/ph19071117

**Published:** 2026-07-20

**Authors:** Sophia Ogechi Ekeuku, Mohammed Farhan Abed Al Salman, Nur Vaizura Mohamad, Sok Kuan Wong, Kok-Yong Chin

**Affiliations:** 1 Department of Pharmacology, Faculty of Medicine, Universiti Kebangsaan Malaysia, Bandar Tun Razak 56000, Malaysia; sogechie@ukm.edu.my (S.O.E.); p115795@siswa.ukm.edu.my (M.F.A.A.S.); sokkuan@ukm.edu.my (S.K.W.); 2 Centre for Drug & Herbal Development, Faculty of Pharmacy, Universiti Kebangsaan Malaysia, Kuala Lumpur 50300, Malaysia; vaizura@ukm.edu.my

**Keywords:** geranylgeraniol, mevalonate pathway, osteoclast, osteoblast, bisphosphonate toxicity

## Abstract

**Background/Objectives:** Geranylgeraniol (GGOH), an isoprenoid intermediate of the mevalonate pathway, regulates bone cell viability and function, particularly by mitigating cellular toxicity induced by nitrogen-containing bisphosphonates (N-BPs). Despite this, its role in osteoporosis remains underexplored. This scoping review synthesises evidence on the effects of GGOH in in vitro and in vivo models of osteoporosis. **Methods:** PubMed, Scopus, and Ovid were searched using GGOH- and osteoporosis-related terms. Primary studies evaluating GGOH exposure in cellular or animal osteoporosis models were eligible. Twenty-nine studies met the inclusion criteria. **Results:** In vitro findings demonstrate that GGOH reverses N-BP-induced depletion of geranylgeranyl pyrophosphate, restoring protein prenylation which is essential for osteoclast and osteoblast survival, cytoskeletal organisation, and differentiation. GGOH reduced osteoclast apoptosis, restored nuclear factor of activated T-cells 1 and carbonic anhydrase II expression, and prevented N-BP-associated suppression of bone resorption. In osteoblasts and mesenchymal stem cells, GGOH improved viability, upregulated osteogenic markers including runt-related transcription factor 2, alkaline phosphatase, collagen type I, and bone morphogenetic proteins, and rescued mineralisation impaired by alendronate or zoledronate. Independent of N-BPs, GGOH exerted divergent effects on osteoclasts, by inhibiting osteoclastogenesis or promoting retinoic acid receptor-mediated bone resorption and attenuating zoledronate protection in vascular calcification settings in a model-specific manner. In vivo, dietary GGOH supplementation improved trabecular and cortical bone parameters and reduced serum C-terminal telopeptide of type I collagen in obese mice, indicating suppression of bone resorption. **Conclusions:** Overall, although GGOH shows osteoprotective potential, its capacity to antagonise N-BP efficacy limits systemic co-administration. Current evidence suggests that local delivery may warrant future investigation as a strategy to mitigate N-BP-induced skeletal toxicity. However, studies evaluating bone tissue exposure, pharmacokinetics, and clinically achievable concentrations are required before this approach can be translated.

## 1. Introduction

Osteoporosis is a metabolic bone disorder that presents with decreased bone density and an increased risk of fractures, primarily caused by an imbalance in bone remodelling processes involving osteoblasts and osteoclasts. This imbalance leads to a significant reduction in bone mineral density (BMD) and structural integrity, increasing the risk of fractures [1,2]. This condition primarily affects the elderly and poses significant global health challenges, such as increased morbidity, mortality, and financial burden on healthcare systems [3]. Xiao et al. conducted a systematic review and meta-analysis revealing a global prevalence of osteoporosis at 19.7% [4]. Globally, osteoporotic fractures incur significant healthcare costs, primarily through hospitalisation, emergency care, and outpatient visits. Compared to matched cohorts without fractures, women aged 50 years and older with osteoporotic fractures have been found to face a greater economic burden in countries such as Australia, Germany, and South Korea [5]. Osteoporosis-related fractures in Asia result in more hospital stays and medical care, in which hip fracture care can cost anywhere from USD 774 to USD 14,198.90 [6]. Proactive prevention and cost-effective interventions could lessen the burden of osteoporosis and fractures on the healthcare system.

The mevalonate pathway is a vital metabolic cascade responsible for the biosynthesis of key biomolecules, including cholesterol, ubiquinone, and prenylated proteins. This complex biochemical network produces isoprenoids, which are essential for regulating diverse cellular functions and processes [7,8]. The mevalonate pathway is critical for regulating bone metabolism, especially in the context of osteoporosis. This pathway is critical to produce farnesyl pyrophosphate and geranylgeranyl pyrophosphate (GGPP), which are required for protein prenylation and influence the activity of osteoclasts and osteoblasts [9]. Disruption of this pathway can cause an imbalance in bone remodelling, favouring bone resorption over formation, which is characteristic of osteoporosis. Bisphosphonates can inhibit this pathway, reducing protein prenylation, affecting cell viability and function and potentially leading to conditions such as medication-related osteonecrosis of the jaw (MRONJ) [10]. Statins, which also influence this pathway, have shown promise in increasing bone mineral density and preventing bone loss. However, prolonged use of statins has been linked to increased diabetes and liver enzymes, as well as medical conditions affecting the muscle (myopathy, myalgia, and rhabdomyolysis), diabetes mellitus, and central nervous system [9]. While disrupting the mevalonate pathway can lead to osteoporosis, it also offers opportunities for therapeutic intervention.

Geranylgeraniol (GGOH) is a naturally occurring isoprenoid alcohol that serves as a crucial component of the mevalonate pathway. It is synthesised from GGPP, a vital intermediate in this metabolic pathway. GGOH serves as a key metabolic intermediate, providing a precursor for GGPP, which is necessary for the prenylation of small GTP-binding proteins [11,12]. This post-translational modification is essential for various protein functions, including membrane localisation and the mediation of protein–protein interactions [13,14]. Moreover, GGOH is an intermediate in the mevalonate pathway, which drives cholesterol biosynthesis, which is a process critical for the formation of cellular membranes and the production of steroid hormones and bile acids [7,8]. Additionally, GGOH is involved in the synthesis of ubiquinone, a coenzyme that plays an indispensable role in mitochondrial electron transport [8]. GGOH has been shown to suppress osteoclast differentiation while enhancing osteoblast differentiation in primary cultures of bone marrow and calvarial cells, respectively [15]. The objective of this scoping review is to examine the existing evidence on the role of GGOH in bone metabolism and its potential implications for the prevention and treatment of osteoporosis. A scoping review was chosen because the evidence on GGOH in osteoporosis is diverse and exploratory, necessitating the mapping of cellular mechanisms, skeletal outcomes, and knowledge gaps across multiple experimental models. By consolidating current research findings, this review aims to deliver a comprehensive analysis of the association between GGOH and osteoporosis, while identifying areas for future investigation and clinical translation. Additionally, it may offer critical insights to researchers and healthcare practitioners regarding the potential of GGOH as an emerging therapeutic strategy for osteoporosis management.

## 2. Materials and Methods

This scoping review was conducted in accordance with the guidelines established by Arksey and O’Malley [16] and adhered to the PRISMA checklist for Scoping Reviews [17]. The procedure entails formulating the research question, identifying pertinent studies, selecting suitable studies, extracting data, and subsequently synthesising, summarising, and reporting the results. The protocol of this scoping review has been registered in the Open Science Framework (url: https://osf.io/rnqs7/) (assessed on 17 July 2026).

### 2.1. Identifying the Research Question

This scoping review was conducted to address the research question: “What are the effects of GGOH supplementation on osteoporosis?” The inquiry was constructed utilising the Population–Intervention–Comparator–Outcome (PICO) framework. The population was characterised as cell lines or animal models of bone loss, or human patients diagnosed with osteopenia or osteoporosis. The concept was GGOH supplementation, and the context encompassed any outcomes relevant to skeletal health.

### 2.2. Identifying Relevant Studies

A systematic literature search was conducted in November 2025 using the PubMed, Scopus, and Ovid databases. The search strategy used the terms (“Geranylgeraniol”) AND (“bone” OR “osteoporosis” OR “osteoclast” OR “osteoblast”) in article titles and abstracts to retrieve relevant records from database inception until November 2025. No restrictions on publication year were applied. The complete search strategies for each database are provided in Supplementary Table S1.

Eligibility criteria were developed using the PICO framework (Table 1).

#### 2.2.1. Other Inclusion Criteria

- Articles containing primary data (in vitro, animal, or clinical studies).
- Studies with the primary objective of evaluating the effects of GGOH on bone-related cells, tissues, or skeletal outcomes.
- Articles published in English.
- Full-text articles available.

#### 2.2.2. Exclusion Criteria

- Reviews, systematic reviews, meta-analyses, editorials, commentaries, conference abstracts, and opinion papers.
- Articles without primary data.
- Studies investigating vitamin K_2_ supplementation without evaluating the effects of GGOH.
- Studies in which the effects of GGOH could not be distinguished because of combination therapies.
- Studies investigating bisphosphonate- or medication-related osteonecrosis of the jaw (BRONJ/MRONJ), unless they included experiments evaluating the direct effects of GGOH on osteoblastic or osteogenic cell models relevant to the objectives of this review.
- Articles for which the full text could not be obtained.

### 2.3. Study Selection

**Figure 1:**
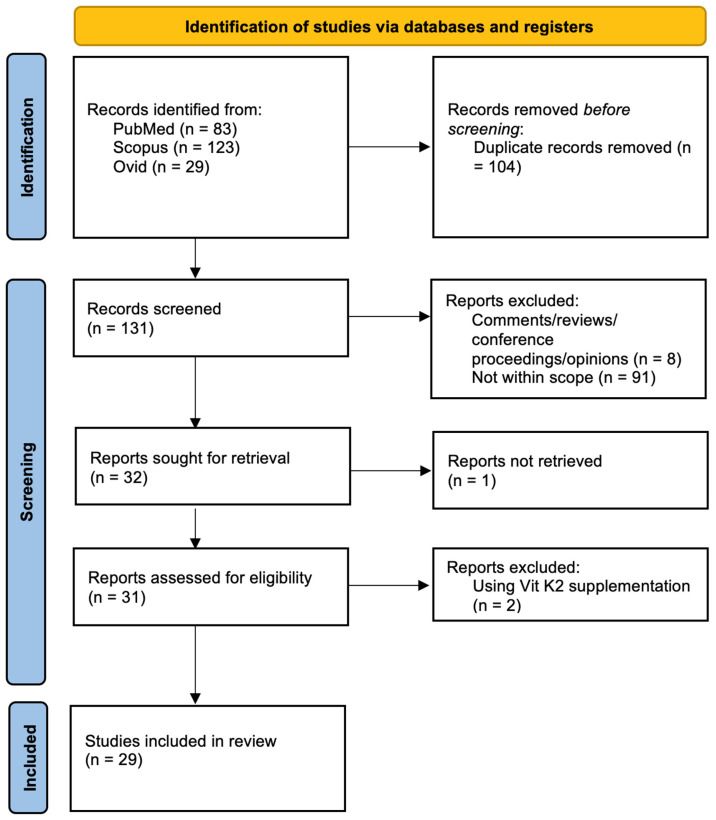
PRISMA flow chart (adapted from https://www.prisma-statement.org/prisma-2020-flow-diagram assessed on 17 July 2026).

The references were organised using Mendeley (version 2.139.0) (Elsevier, London, UK). After consolidating the search results of the three databases, duplicates were removed utilising Mendeley, followed by a manual review to ensure accuracy. Two reviewers (S.O.E. and M.F.A.A.S.) independently screened the titles and abstracts of all retrieved records for eligibility. Articles considered potentially relevant underwent independent full-text assessment by the same reviewers using the predefined eligibility criteria. Any disagreements during either screening stage were resolved through discussion and, when consensus could not be reached, by consultation with a third reviewer (K.-Y.C.). The study selection process is documented in Figure 1. The complete list of excluded and included studies can be found in Tables S2 and S3.

### 2.4. Charting the Data

A standardised Microsoft Excel spreadsheet was developed to extract data from the included studies. The extracted information comprised bibliographic details (author and publication year), study design, experimental model (cell line, explant, animal model or human participants), intervention characteristics (GGOH concentration or dose, treatment duration and comparator where applicable), skeletal outcomes assessed, molecular mechanisms investigated, principal findings, study limitations, and authors’ conclusions. Data extraction was performed by S.O.E. and independently verified by N.V.M. and S.K.W.

### 2.5. Collating, Summarising, and Reporting the Results

A qualitative synthesis of the data was conducted, with findings presented in accordance with principal pathological features of osteoporosis, including bone resorption indices, structural parameters, and metabolic markers of bone. The significant variability in study designs, animal models, and reported outcomes prevented a quantitative statistical synthesis of the data. The ensuing discussion detailed the function of GGOH in the pathogenic cascade of osteoporosis, existing knowledge deficiencies, and the intrinsic limitations of the current review.

## 3. Results

Using the predefined PCC-based eligibility criteria, the systematic search identified 235 unique records. After screening against the eligibility criteria, 104 articles were excluded. The grounds for exclusion were irrelevance of topic (*n* = 91), publication type such as reviews, conference abstracts, comments, or opinion papers (*n* = 8), a focus on vitamin K2 supplementation (*n* = 2), and unavailability of the full text (*n* = 1). Thus, a total of twenty-nine articles were selected for final inclusion. The procedure for article screening and selection is summarised in Figure 1.

### 3.1. Study Characteristics

The studies utilised a mix of in vitro (twenty-seven studies focusing on cell lines or explants) and in vivo (two studies focusing on dietary intervention in mice) animal models. No clinical studies were reported on this topic.

In vitro models used a variety of cell types and explants to study osteoclast, osteoblast, and chondrocyte function. Osteoclast-related studies utilised murine osteoclastogenesis cocultures using murine spleen cells with murine stromal cells TMS-14 [18] or TMS-12 [19] and MB1.8 osteoblasts with bone marrow cells [20,21]. Other cell types utilised for osteoclast-related studies include purified murine osteoclasts (90–95% purity) for kinase assays, and rabbit osteoclast bone resorption assays utilising bovine bone slices [20], J774 macrophage cells [22], human mononuclear cells [23], RAW264.7 cells [24], mouse osteoclast precursors [25], human osteoclast precursors [10,11], and human peripheral blood mononuclear cells [26]. Explant models included metatarsal explants [27] and metatarsal explants prelabelled with calcium-45 (45Ca) [28,29,30]. Osteoblast/chondrocyte studies utilised bone marrow cells (BMC) [31], pre-osteoblast MC3T3-E1 cells [32,33,34,35], human osteogenic cells [36,37], human osteoblast cells (hoBs) [10,11,38], human bone marrow mesenchymal stem cells (hMSCs) [39], human osteoblastic osteosarcoma MG-63 cells [40], aortic thoracic vascular smooth muscle cells (VSMCs, A7r5) [41], and primary endochondral chondrocytes isolated from costochondral growth plates of male C57BL/6J mice [42]. The in vivo studies used male C57BL/6J mice. Obesity and associated disorders, such as impaired glucose homeostasis, were induced by feeding a high-fat diet (HFD) (60% of calories from fat) [43,44].

The compound investigated for both in vitro and in vivo studies was GGOH. In vitro dosages of GGOH ranged from 10 µM to 100 µM and treatment duration was between 1 h and 28 days. For in vivo studies, GGOH was supplemented in the diet at 400 and 800 mg/kg diet, and the intervention lasted for 14 weeks.

### 3.2. Skeletal Effects of GGOH from In Vitro Studies

#### 3.2.1. Effects on Osteoclasts

GGOH influenced osteoclast biology through both indirect and direct mechanisms. Indirectly, GGOH counteracted the inhibitory and pro-apoptotic effects of nitrogen-containing bisphosphonates (N-BPs) by restoring the mevalonate pathway. Directly, in the absence of N-BPs, GGOH exerted independent effects on osteoclast differentiation and resorptive activity through distinct signalling pathways.

GGOH specifically counteracted the inhibitory and pro-apoptotic effects of N-BPs. It inhibited apoptosis induced by alendronate and risedronate in isolated murine osteoclasts, but not apoptosis induced by non-nitrogen-containing bisphosphonates such as etidronate or clodronate, and significantly reduced amino-bisphosphonate-induced caspase-3 activity [20,21,22,28,29,31]. GGOH restored alendronate-inhibited bone resorption in rabbit osteoclasts [20], reversed zoledronate-induced suppression of acid-activated chloride currents [24], restored nuclear factor of activated T-cells 1 (NFATc1) and carbonic anhydrase II (CAII) expression in osteoclast precursors [25], enhanced zoledronate-suppressed osteoclast differentiation [11], and prevented disruption of osteoclastic actin rings and cytoskeletal organisation [19,21].

In the absence of N-BPs, GGOH exerted independent effects on osteoclast biology. Hara et al. [18] and Hiruma et al. [19] demonstrated that GGOH inhibited osteoclastogenesis by reducing tartrate-resistant acid phosphatase (TRAP) activity, decreasing TRAP-positive multinucleated cell formation, and suppressing the receptor activator of nuclear factor kappa-B ligand (RANKL) mRNA expression in models stimulated with 1,25-dihydroxyvitamin D_3_ (1,25(OH)_2_D_3_) or prostaglandin E2 (PGE_2_). Conversely, Van Beek et al. [30] reported that GGOH directly stimulated resorption by mature osteoclasts through a retinoic acid receptor (RAR)-dependent mechanism, indicating that its effects may vary according to the differentiation stage of osteoclasts and the signalling pathways involved.

#### 3.2.2. Effects on Osteoblasts

GGOH demonstrated a dual, context-dependent impact on osteoblasts, acting as a robust cytoprotective agent against N-BP toxicity, while possibly hindering differentiation when used in isolation.

Under the N-BP challenge, GGOH consistently served as a robust safeguard for cellular viability. It safeguarded MC3T3-E1 pre-osteoblasts from apoptosis caused by the geranylgeranyl transferase I inhibitor GGTI-2166 [32], inhibited the increment of p27Kip1 in MC3T3-E1 cells [33] and markedly diminished the suppressive effects of alendronate and zoledronate on the viability of human osteoblasts [10,11,36,37]. This cytoprotection was concentration-dependent, with low to moderate concentrations of GGOH (10–40 µM) not only counteracting the effects of zoledronate in hMSCs [39], but also increasing viability in human osteoblasts up to 150% [10]. A high concentration of GGOH (80 µM) synergistically enhanced the efficacy of zoledronate in the viability of hMSCs and human osteoblasts [10,39]. When used alone at high concentration, GGOH (50–100 µM) significantly impeded cell viability in human osteogenic cells but effectively reinstated osteogenic capacity inhibited by zoledronic acid [36]. GGOH counteracted the inhibitory effects of alendronate on MC3T3-E1 cell functionality and mineralisation [34,35]. It abolished alendronate-altered MC3T3-E1 morphology, decreased cell area, actin stress fibre density, and nuclear area [34]. GGOH also restored compromised actin networks and enhanced the mineralisation potential of zoledronate-treated MG-63 cells when incorporated into collagen membranes [40]. The restorative impact on mineralisation is most pronounced when GGOH is administered during the initial osteogenic phase (days 0–7) [35]. This functional recovery is mechanistically supported by the reversal of N-BP-suppressed gene expression, as GGOH reinstated levels of critical markers such as alkaline phosphatase (ALP), type I collagen (COL1), runt-related transcription factor 2 (RUNX2), osteopontin (OPN), vascular endothelial growth factor (VEGF), VEGF receptor 2 (VEGFR2), osteoprotegerin (OPG), osteocalcin (OSC), and bone morphogenic protein 2/7 (BMP-2/7) [11,33,35,40,41]. Overall, the effects of GGOH on osteoblasts were highly dependent on experimental conditions. In most studies, GGOH primarily acted as a cytoprotective agent by reversing N-BP-induced impairment of osteoblast viability, differentiation, and mineralisation. However, when administered alone, the response was concentration-dependent, with lower concentrations generally supporting cell viability, whereas higher concentrations (50–100 µM) reduced viability in some osteogenic cell models.

#### 3.2.3. Effects on Chondrocytes and Other Cells

GGOH has intricate and frequently contradictory effects on chondrocytes and, more generally, on tissue models of vascular calcification and angiogenesis. The ability of growth plate chondrocytes to counteract alendronate’s effects on VEGF has been the subject of research. In this case, GGOH did not reverse the reduction in secreted VEGF protein brought on by a high alendronate concentration, while VEGF mRNA isoform levels were restored by GGOH [42]. GGOH also blocked the inhibitory effects of alendronate and risendronate on total fibroblastic colony formation but did not reverse the stimulation of total colony numbers [31]. Additionally, the antiangiogenic ligands C-X-C motif chemokine ligand 9 and 10 (CXCL9 and 10), which are upregulated by zoledronic acid, were significantly downregulated in primary human osteoclasts upon co-addition of GGOH, possibly re-establishing angiogenic function [26]. On the other hand, GGOH negates zoledronate’s protective effects in a model of pathological vascular calcification using VSMCs going through osteogenic differentiation. When zoledronate and GGOH were co-cultured, the calcification was noticeably higher than when zoledronate was used alone. Additionally, GGOH reversed zoledronate’s regulatory effect on the OPG/RANKL axis by increasing RANKL protein expression and decreasing OPG, thereby creating a pro-calcific environment [41].

#### 3.2.4. Skeletal Effects of GGOH from In Vivo Studies

GGOH exhibited notable osteoprotective effects on bone microarchitecture and quality in an obese murine model. In trabecular bone at the fourth lumbar vertebra (LV-4), GGOH administered at 800 mg/kg diet enhanced overall microarchitecture [44]. A reduced dosage of 400 mg/kg diet notably enhanced trabecular number (Tb.N) and connectivity density (Conn.Dn), while diminishing trabecular separation (Tb.Sp) and the structure model index (SMI), signifying a transition towards a more plate-like and resilient trabecular architecture [43]. At the femoral midshaft, GGOH supplementation at 800 mg/kg diet increased cortical bone volume fraction (BV/TV) and stiffness relative to the HFD control group, indicating improved cortical bone mass and mechanical integrity [44]. Analysis of bone turnover biomarkers indicated that GGOH supplementation at doses of 400 mg/kg and 800 mg/kg reduced serum levels of the bone resorption marker C-terminal telopeptide of type I collagen (CTX-1), suggesting a systemic anti-resorptive effect in the obese model. Conversely, GGOH did not exert a significant influence on the bone formation marker procollagen type 1 N-terminal propeptide (P1NP) [43,44]. Table 2 summarises the study designs and principal findings of the included studies.

### 3.3. Mechanism of Action

The impact of GGOH on bone parameters is determined by two primary molecular mechanisms, depending on its function as an antagonist of N-BP toxicity or as an independent modulator. The fundamental mechanism involves circumventing the N-BP-induced inhibition of the mevalonate pathway. N-BPs inhibit farnesyl pyrophosphate synthase (FPPS), resulting in the depletion of GGPP, which is crucial for the prenylation of small GTP-binding proteins (e.g., Rho, Rac, Cdc42) that regulate cytoskeletal integrity, vesicular trafficking, and cell survival. GGOH replenishes this essential metabolic pool by acting as a substrate for intracellular conversion to GGPP [45]. This restoration underpins the cytoprotection of osteoblasts and mesenchymal stem cells [10,11,34,35,39], the reversal of osteoclast apoptosis and kinase activation (Mst1) [20,21,29], the recuperation of osteogenic gene expression (RUNX2, ALP, COL1), and mineralisation [11,33,35,40], as well as the reversal of N-BP-induced antiangiogenic factors (CXCL9/10) in osteoclasts [26].

A key mechanistic finding emerging from the available evidence is the selective ability of GGOH to reverse the effects of N-BPs, while having no effect on non-N-BPs. N-BPs inhibit farnesyl pyrophosphate synthase in the mevalonate pathway, resulting in depletion of GGPP and impaired geranylgeranylation of small GTPases required for osteoclast survival and cytoskeletal function. Supplementation with GGOH restores intracellular GGPP pools, rescues protein prenylation, and reverses osteoclast dysfunction and apoptosis [46]. Conversely, non-N-BPs induce osteoclast apoptosis through the intracellular generation of non-hydrolysable ATP analogues, a mechanism independent of protein prenylation [47]. Accordingly, GGOH does not reverse the effects of these agents. This differential response provides compelling evidence that the primary mechanism of GGOH may be the restoration of GGPP-mediated protein prenylation rather than a non-specific cytoprotective effect.

**Figure 2:**
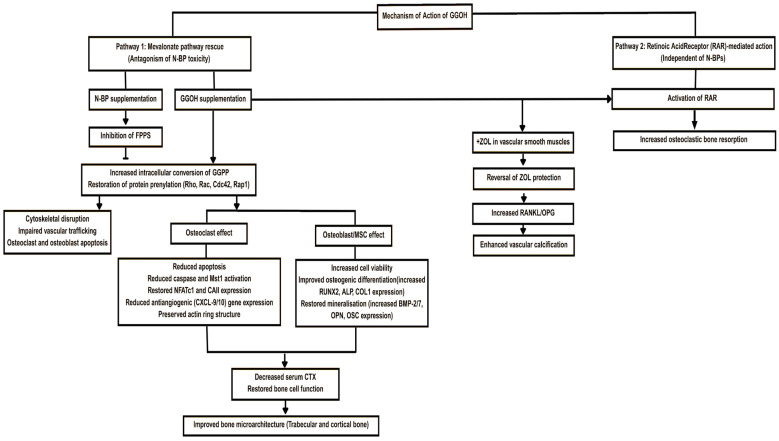
Dual molecular pathways mediating the effects of geranylgeraniol on bone.

Additionally, the RAR pathway operates via an independent mechanism: GGOH or its metabolite, geranylgeranoic acid (GGA), directly induces bone resorption, which RAR antagonists successfully inhibit [30]. A complex interaction is demonstrated by the overall in vivo outcome of these actions, which includes increased cortical stiffness and enhanced trabecular architecture, accompanied by reduced serum CTX. Nonetheless, this mechanism can be harmful in non-skeletal contexts; in vascular smooth muscle cells, GGOH undermines zoledronate’s protective effect against calcification by fostering a pro-calcific state through the modulation of the RANKL/OPG ratio [41]. Consequently, although GGOH primarily restores crucial protein prenylation disrupted by N-BPs, its multifaceted effects underscore a context-dependent duality, providing therapeutic relief for N-BP-compromised bone cells while potentially aggravating pathological processes in the vasculature. Figure 2 summarises the mechanism of action of GGOH.

## 4. Discussion

GGOH is particularly significant in bone metabolism because it counteracts the molecular effects of N-BPs, which are first-line therapies for osteoporosis. Mechanistically, N-BPs inhibit the mevalonate pathway, leading to depletion of geranylgeranyl pyrophosphate (GGPP), impaired protein prenylation, and subsequent cellular dysfunction and apoptosis in both osteoclasts and osteoblasts. GGOH directly addresses this blockade by serving as a precursor for GGPP, thereby restoring the geranylgeranylation of small GTP-binding proteins, including RhoA and Rap1A/B [48,49]. This restoration rescues osteoclasts from N-BP-induced inhibition [20,21,29] and preserves osteoblast viability and osteogenic function under conditions of mevalonate pathway inhibition [10,11,34,35,38]. In addition, GGOH’s ability to restore fibroblast viability and function suggests a potential role in promoting wound healing, which is frequently impaired during bisphosphonate therapy [31]. Importantly, no clinical studies evaluating GGOH in osteoporosis or other skeletal disorders were identified. Therefore, all conclusions regarding the osteoprotective potential of GGOH are derived exclusively from preclinical evidence and should be interpreted cautiously until validated in human studies.

An important consideration for the therapeutic application of GGOH is its dual mechanism of action, which carries significant clinical implications. Restoration of the mevalonate pathway underlies its ability to mitigate N-BP-induced cytotoxicity by replenishing GGPP and restoring protein prenylation. However, the same mechanism may also diminish the intended anti-resorptive effects of N-BPs by restoring osteoclast function. This duality creates a fundamental therapeutic dilemma. While GGOH may alleviate N-BP-associated skeletal toxicity, systemic administration during osteoporosis treatment could potentially compromise the efficacy of anti-resorptive therapy. This is supported by studies demonstrating that GGOH reverses N-BP-induced inhibition of bone resorption in explant models [27,28,29] and attenuates zoledronate’s protective effects against vascular calcification by modulating the OPG/RANKL ratio [41]. Consequently, the therapeutic value of GGOH is highly dependent on the clinical context, and systemic co-administration with N-BPs should be approached with caution.

The apparent divergent effects of GGOH on osteoclasts could be explained by differences in experimental context rather than intrinsically opposing biological actions. Studies evaluating 1,25(OH)_2_D_3_- or PGE_2_-induced osteoclastogenesis demonstrated that GGOH inhibited osteoclast differentiation, whereas studies using zoledronate-treated cells showed that zoledronate restored physiological osteoclast differentiation by replenishing GGPP following mevalonate pathway inhibition. In contrast, Van Beek et al. [30] investigated mature osteoclasts and demonstrated increased resorptive activity mediated through retinoic acid receptor (RAR) signalling. Collectively, these findings suggest that the biological effects of GGOH on osteoclasts are influenced by the initiating stimulus, the stage of osteoclast differentiation, and the signalling pathways involved, rather than representing inherently contradictory actions.

Notwithstanding the potential limitations of systemic co-therapy, the available in vivo evidence suggests that GGOH possesses osteoprotective properties in metabolically compromised bone. Although the two in vivo studies were conducted in high-fat diet (HFD)-induced obese mice rather than in classic osteoporosis models, obesity-induced bone deterioration shares several pathogenic mechanisms with osteoporosis, including chronic low-grade inflammation, oxidative stress, impaired osteoblast differentiation, increased bone marrow adiposity, and dysregulated bone remodelling [50]. Consequently, HFD-induced obesity provides a useful model for investigating interventions targeting metabolic bone impairment. Dietary GGOH supplementation improved glucose homeostasis, reduced systemic inflammation, enhanced trabecular microarchitecture (increased Tb.N and decreased SMI), and lowered serum CTX-1 concentrations, suggesting preservation of bone quality. These effects may be mediated, at least in part, through inhibition of NF-κB signalling and attenuation of chronic inflammation [43,44]. Nevertheless, these findings should be interpreted as mechanistic evidence from a metabolically compromised bone model rather than as direct evidence of efficacy in classical osteoporosis, as ageing- and oestrogen-deficiency-induced bone loss involves additional endocrine and cellular mechanisms.

Although local delivery has been proposed as a potential strategy to minimise systemic interference with N-BP therapy, its clinical feasibility remains uncertain. Currently, no pharmacokinetic studies have characterised the absorption, biodistribution, bone tissue penetration, local retention, or clearance of GGOH following either systemic or local administration. Consequently, it remains unknown whether the cytoprotective concentrations commonly employed in vitro (50–100 µM) are pharmacologically achievable within bone tissue under clinically relevant dosing conditions. Future studies should establish the pharmacokinetic profile of GGOH, quantify bone tissue concentrations following different delivery routes, and define the pharmacokinetic–pharmacodynamic relationships required for bone protection before local delivery or other therapeutic strategies can be considered clinically viable.

Several important limitations within the current evidence base restrict the translation of GGOH as a potential therapeutic agent for osteoporosis. First, research has primarily focused on mitigating N-BP-induced cellular toxicity, with most in vitro studies evaluating GGOH’s ability to reverse cellular damage rather than its independent therapeutic potential for osteoporosis or fracture healing. Consequently, the role of GGOH as a primary osteoprotective agent remains poorly defined. Second, the available evidence is dominated by in vitro and ex vivo studies, with only two in vivo investigations identified. Consequently, the translational relevance of the current evidence remains uncertain. Furthermore, no published studies have characterised the pharmacokinetic profile, tissue distribution, or bone concentrations of GGOH following systemic or local administration. This pharmacokinetic uncertainty represents a major barrier to clinical translation and highlights the need for future pharmacokinetic–pharmacodynamic studies to establish clinically relevant dosing strategies. Third, the therapeutic window of GGOH remains incompletely defined. Although low-to-moderate concentrations consistently improved cell viability in several studies, higher concentrations (e.g., 80 µM) reduced cell viability, suggesting a dose-dependent effect that complicates the identification of safe and effective therapeutic dosing. Fourth, an inherent mechanistic challenge exists. While restoration of the mevalonate pathway underlies GGOH’s ability to rescue N-BP-induced cytotoxicity, its concurrent ability to stimulate osteoclastic bone resorption through RAR signalling raises concerns that systemic administration may compromise the anti-resorptive efficacy of N-BPs in patients receiving osteoporosis treatment. Finally, the current in vivo evidence is limited to two studies conducted by the same research group using the same HFD-induced obese mouse model and supported by the same funding source. Consequently, the available preclinical evidence cannot be considered fully independent, and the generalisability of the reported osteoprotective effects remains limited. Independent validation by different research groups using diverse preclinical models, including established ovariectomised and ageing models of osteoporosis, is necessary before robust translational conclusions can be drawn.

The present scoping review also has several methodological limitations. The literature search was restricted to English-language publications indexed in three electronic databases, and grey literature was not searched, potentially introducing publication bias. A formal methodological quality assessment and risk-of-bias evaluation of the included studies was not performed. Consequently, the findings presented in this review should be interpreted as a mapping of the available evidence rather than an assessment of the certainty or strength of that evidence. Nevertheless, this review provides a comprehensive synthesis of the current evidence, identifies important mechanistic insights, and highlights key knowledge gaps to guide future preclinical and translational research.

## 5. Conclusions

In summary, GGOH serves as a powerful and specific antagonist of N-BP toxicity by salvaging the mevalonate pathway, thus restoring crucial protein geranylgeranylation and maintaining the viability and function of bone cells. Although dietary GGOH demonstrates potential in enhancing bone microarchitecture in metabolic disease models, its ability to independently induce bone resorption through the RAR pathway and the risk of dose-dependent synergistic toxicity inhibit its systemic application in osteoporosis patients, as this would compromise the primary anti-resorptive effect of N-BP therapy. Thus, the most feasible clinical application for GGOH is as a localised therapeutic agent to alleviate N-BP-related complications. Future research must now focus on translating this potential by validating localised delivery systems, optimising safe dosing parameters and confirming efficacy in rigorous pre-clinical models of N-BP toxicity. Overall, the available preclinical evidence suggests that GGOH possesses promising osteoprotective properties. However, the current evidence base remains limited, particularly in vivo evidence, which is derived from a small number of non-independent studies and limited clinical studies evaluating its efficacy or safety in humans. Therefore, these findings should be considered preliminary, and further independent preclinical studies and well-designed clinical investigations should be conducted before the therapeutic potential of GGOH for osteoporosis can be established.

## Figures and Tables

**Table 1:** Eligibility criteria of the review based on the PICO framework.

Concept	Eligibility Criteria
Population	In vitro studies using osteoblasts, osteoclasts, osteogenic cells, mesenchymal stem cells, chondrocytes, or other bone-related cell models. Animal models of bone loss or impaired bone metabolism. Human participants with osteopenia, osteoporosis, or other bone-related disorders (if available).
Intervention	Exposure or supplementation with GGOH, either alone or in the presence of agents that affect the mevalonate pathway (e.g., nitrogen-containing bisphosphonates).
Comparator	Negative/Vehicle/Placebo control group.
Outcome	Outcomes related to skeletal health, including osteoblast or osteoclast viability and function, bone remodelling, bone turnover, bone mineral density, bone microarchitecture, bone strength, mineralisation, or fracture healing.

**Table 2:** Effect of GGOH on cells and animals.

Researcher	Study Design	Major Findings
Hara et al. [18]	Co-culture of murine spleen cells and murine stromal cells (TMS-14) in the presence of 1,25(OH)_2_D_3_ treated with GGOH (1–10 µM) for 7 days.	GGOH ↓TRAP activity and TRAP + MNC formation vs. control
Fisher et al. [20]	Coculture of MB 1.8 osteoblastic cells and mouse marrow cells in the presence of 1,25(OH)_2_D_3_ and treated with or without GGOH (10 µM)/lovastatin (10 µM)/alendronate (10, 15, 60 µM) on day 5 and 6 of 7-day culture. Osteoclasts culture from the tibiae of New Zealand White rabbits plated onto bovine bone slices, in the presence of 1,25(OH)_2_D_3_ and incubated with lovastatin (10 µM)/alendronate (15 and 60 µM) with or without GGOH (10 µM) for 3 days.	GGOH ↑ TRAP+ cells inhibited by alendronate and lovastatin inhibition of osteoclast formation and resorption GGOH blocked alendronate and lovastatin-mediated activation of 34 kDa kinase
Benford et al. [22]	J774 macrophage cell incubated with amino bisphosphonates (alendronate, ibandronate, pamidronate) with or without GGOH (50 µM) for 24 h.	GGOH ↓ apoptosis of J774 macrophages induced by amino bisphosphonates (ibandronate/pamidronate) GGOH ↓ caspase 3-like activity induced by amino bisphosphonates (alendronate/ibandronate)
Van Beek et al. [28]	Osteoclast culture of foetal metatarsal explants pre-labelled with ^45^Ca and incubated with bisphosphonate and mevastatin with or without GGOH (0–100 µM) for 3 days.	GGOH alone ↑ osteoclastic resorption at low concentration (50 and 100 µM) but ↓ at high dose (500 µM) GGOH (50 and 100 µM) ↑ mevastatin-inhibited ^45^Ca release GGOH (50 and 100 µM) ↑ ^45^Ca release inhibited by nitrogen-containing bisphosphonates; ↔ on ^45^Ca release inhibited by non-nitrogen-containing bisphosphonates ↑ TRAP+ osteoclast in GGOH (50 µM) −/+ ibandronate ↔ TRAP+ osteoclast in GGOH (50 µM) + clodronate
Reszka et al. [21]	Murine bone marrow cells in the presence of 1,25(OH)_2_D_3_ cocultured with MB 1.8 osteoblastic cells. Osteoblasts removed upon osteoclast formation. Osteoclasts cells were maintained with M-CSF and treated with bisphosphonates/lovastatin with or without GGOH (10 µM).	GGOH ↓ alendronate/risendronate-induced osteoclast apoptosis; ↔ clodronate/etidronate -induced osteoclast apoptosis GGOH ↓ both nuclear condensation and loss of the actin ring structure in alendronate/risendronate-treated osteoclasts but not clodronate/etidronate-treated osteoclasts GGOH ↓ Mst1 cleavage induced by alendronate, risedronate and lovastatin but not clodronate
Van Beek et al. [27]	Osteoclast culture from foetal mouse metatarsal in the presence of ^45^Ca and Na β-glycerophosphate and treated bisphosphonates +/− GGOH (100 µM) + or 1–2 days.	GGOH ↑ alendronate-inhibited ^45^Ca; ↔ on clodronate-inhibited ^45^Ca release
Van Beek et al. [29]	Osteoclasts from foetal mouse metatarsal explants pre-labelled with 30 Ci ^45^Ca and treated bisphosphonates +/− GGOH (100 µM) + or 1–2 days.	GGOH ↑ EC 50 for bone resorption inhibition of alendronate and risedronate by 100-fold GGOH partially ↑ EC 50 for bone resorption inhibition of pamidronate by 10-fold
Still et al. [31]	Rat bone marrow stromal cell treated with bisphosphonate +/− (1000 µM) for 1–2 days.	GGOH ↓ inhibitory effect of alendronate/risedronate (10 µM) on total fibroblastic colony formation GGOH did not reverse the stimulation of total colony numbers caused by the addition alendronate/risedronate (0.01 µM)
Taira et al. [23]	Human mononuclear cells isolated from peripheral blood of 10 normal, healthy subjects. In the presence of RANKL and human M-CSF and treated +/− GGOH (0.1–10 µM) for 7–14 days.	GGOH (1–10 µM) ↓ TRAP+ MNCs and percentage area of lacunae resorption
Hiruma et al. [19]	Co-culture system of spleen cells and TMS-12 stromal cells in the presence of RANKL, PGE, and 1,25(OH)_2_D_3_ and treated with nitrogen-containing bisphosphonates +/− GGOH (5 µM) for 1, 2, 3, 4, 5, and 7 days.	GGOH ↓ 1,25(OH)_2_D_3_-induced TRAP+ MNCs; ↔ on 1,25(OH)_2_D_3_-induced PGE_2_ production GGOH ↓ PGE-induced TRAP+ MNCs GGOH ↓ 1,25(OH)_2_D_3_-induced mRNA expression of RANKL GGOH ↓ alendronate/risedronate-induced disruption of osteoclastic actin rings
Yoshida et al. [33]	Mouse monoclonal pre-osteoblast MC3T3-E1 cells treated with GGOH (10 uM) for 7 days.	GGOH treatment ↓the increment of p27^Kip1^ induced by cell-to-cell contact in MC3T3- E1 cells GGOH ↑ protein expressions of ALP, BMP-2, and extracellular mineralisation
Van Beek et al. [30]	Osteoclast culture from fetal mouse metatarsal explants pre-labelled with 30 Ci ^45^Ca and treated with ibandronate +/− GGOH (1–100 uM) for 1–2 days.	GGOH (1–100 µM) alone ↑ %^45^Ca release in a dose dependent manner; GGOH (100 µM) ↑ ibandronate-induced suppression of %^45^Ca release GGOH ↑ resorption of the mineralised bone matrix and reduced the size of whole bone explant, including the cartilage ends vs. control GGOH ↑ RARβ mRNA expression in bone explants
Evans and Oberbauer [42]	Primary chondrocytes isolated from costochondral growth plates of C57BL/6J male mice treated with alendronate +/− GGOH (10 µM) for 24 h.	GGOH partially to completely ↑ alendronate-induced reduction in VEGF 120 and 164 mRNA expression level GGOH further ↓ alendronate-induced reduction in media and cell VEGF protein expression level
Yoshida et al. [32]	MC3T3-E1 pre-osteoblastic cells treated with geranylgeranyl transferase I inhibitor/Rho transferase inhibitor +/− GGOH (10 µM).	GGOH ↓ caspase-3 activity and cell death induced by geranylgeranyl transferase I inhibitor but not Rho kinase inhibitor
Ziebart et al. [37]	Human osteogenic cells treated cultured with bisphosphonates +/− GGOH (10 µM) for 72 h.	GGOH ↑ the viability suppressed by ibandronate, pamidronate, zoledronate GGOH ↑ cell migration suppressed by zoledronate GGOH reversed cytoskeletal distortion in all cells caused by ibandronate, pamidronate, zoledronate
Ohgi et al. [24]	Osteoclasts derived from RAW264.7 cells in the presence of RANKL and murine bone marrow macrophages from tibia and femora of ddY male mice in the presence RANKL and M-CSF treated with zoledronic acid +/− GGOH (30 µM).	GGOH ↑ outward and inward Cl^−^ current suppressed by zoledronic acid in an acidic environment GGOH alone had ↔ on the acid-activated Cl^−^ currents in osteoclasts
Nakagawa et al. [25]	Osteoclast precursors cells from ICR mice +/− RANKL/zoledronate/GGOH (100 µM) for 2 days.	GGOH ↑ multinucleated cells; NFATc1 and carbonic anhydrase II expression suppressed by zoledronate
Hagelauer et al. [36]	Human osteogenic cells treated with zoledronate +/− GGOH (0–100 µM).	GGOH (50–100 µM) alone ↓ cell viability; ↔ on migration GGOH (50–100 µM) ↑ cell viability, migration and wound healing suppressed by zoledronate GGOH reversed actin skeleton disorganisation induced by zoledronate
Fliefel et al. [10]	Human osteoblast and osteoclast treated with zoledronate +/− or GGOH (10–80 µM).	GGOH alone (10, 20, and 40 µM) ↑ cell viability; GGOH (80 µM) alone ↓ viability in human osteoblasts +/− zoledronate GGOH (10 µM) ↑ zoledronate-induced suppression of Rap 1A/B expression in both osteoblasts and osteoclasts
Zafar et al. [26]	Human osteoclast cells generated from peripheral blood mononuclear cells of three healthy post-menopausal women in the presence of M-CSF and RANKL and treated with zoledronate +/− GGOH (50 µM) for 2 days.	GGOH ↓ zoledronate-induced increase in antiangiogenic genes (CXCL9, CXCL10) and TNF GGOH ↓ zoledronate-induced increase in CCL2, TGFBR1, ENG, and CXCL1 Osteoclasts cultured with GGOH + zoledronate showed both large multinucleate cells and smaller less defined cells.
Fliefel et al. [39]	Human mesenchymal stem cells incubated with zoledronate +/− GGOH (10–80 µM) 7 days.	GGOH (10–40 µM) ↑; GGOH (80 µM) ↓ cell viability +/− zoledronate
Patntirapong et al. [34]	MC3T3 pre-osteoblast cells incubated with alendronate +/− GGOH (10 and 50 μM) for 3 days.	GGOH (50 μM) partially ↑ alendronate-induced reduction in cell viability, bone nodule formation, cell area, cell perimeter, stress fibre density, mean fibre intensity, nucleus area, nucleus perimeter, and nucleus ratio GGOH (50 μM) partially) ↓ alendronate-induced late cell apoptosis and G2/M cell arrest (eight-fold) GGOH ↓ alendronate-induced cell and nucleus circularity, and mean nuclear intensity
Mungpayabarn and Patntirapong [35]	MC3T3 pre-osteoblast cells incubated with alendronate +/− GGOH (50 μM) for 21 days.	GGOH ↑ mineralisation (day 0–7) vs. alendronate alone and later addition periods GGOH partially ↑ alendronate-reduced cell viability (43% increase) and ↑ total protein content (40% higher) GGOH ↑ gene expressions of COL1, OPN, VEGF, and VEGFR2 (especially at day 3 and 7) vs. alendronate alone
Otto et al. [38]	Human osteoblasts incubated with bisphosphonates +/−GGOH (50 μM) for 21 days.	GGOH ↑ bisphosphonate-induced suppression of cell migration
Jung et al. [11]	Human osteoblasts and human osteoclast precursors in the presence of RANKL and M-CSF incubated with bisphosphonates +/− GGOH (50 μM) for 21 days.	GGOH ↑ cell viability in the alendronate (50 μM) and zoledronate (10 and 50 μM) groups in osteoblasts; clodronate, alendronate, and zoledronate (50 μM) groups in osteoclasts GGOH + zoledronate (50 μM) ↑ osteoclast differentiation vs. zoledronate (50 μM) alone GGOH↑ zoledronate-induced suppression of the ALP, RANKL and type 1 collagen gene expression; alendronate and zoledronate-induced suppression of RUNX2 and OC expression in osteoblasts GGOH ↑ zoledronate-induced suppression of cFOS expression in osteoclast GGOH ↓ zoledronate-induced increase in CALCR gene expression in osteoclasts
Xu et al. [41]	Murin vascular smooth muscle cells incubated with β-glycerophosphate and treated with zoledronate +/− GGOH (30 μM).	GGOH ↑ zoledronate (5 μM)-induced suppression in calcification and calcium content GGOH ↑ zoledronate (5 μM)-induced suppression RANKL, RUNX2 and OPN protein expression; ↓ zoledronate (5 μM)-induced increase in OPG protein expression
Manzano-Moreno et al. [40]	Human osteoblastic osteosarcoma MG-63 cells incubated with β-glycerophosphate and seeded into collagen membranes doped with GGOH (30 μM) +/− zoledronate for 48 h.	GGOH ↑ zoledronate-induced suppression of osteoblast proliferation (two-fold) and mineralisation GGOH ↓ zoledronate-induced increase in OPG/RANKL ratio GGOH ↑ zoledronate-induced suppression TGF-β1, R1, R2, R3, RANKL, OPG, OSX, ALP, RUNX-2, OSC, COL-1, BMP-2, -7, VEGF gene expression GGOH-doped membranes restored zoledronate-treated osteoblast morphology GGOH alone ↑ OSX, VEGF, ↓ ALP gene expression
Chung et al. [44]	Male C57BL/6J mice with HFD-induced obesity +/− GGOH (800 mg/kg) for 14 weeks.	GGOH ↑ glucose tolerance and insulin sensitivity; ↓ CTX GGOH ↑ stiffness, cortical thickness, BV/TV, Conn.Dn; ↓ SMI
Shen et al. [43]	Male C57BL/6J mice with HFD-induced obesity +/− GGOH (400 mg/kg) for 14 weeks.	GGOH ↑ glucose tolerance and insulin sensitivity; ↓ serum CTX vs. control GGOH ↑ Tb.N.; ↓ Tb.Sp, maximised Conn.Dn, and minimised SMI at LV-4

Abbreviation: 1,25(OH)_2_D_3_, vitamin D3; 45Ca, calcium 45; ALP, alkaline phosphatase; BMP-2, -7, bone morphogenic protein 2 and 7; BV/TV, bone volume fraction; CALCR, calcitonin receptor; Conn.Dn, connectivity density; CTX, c-terminal telopeptide; CXCL9 and 10, chemokine ligand 9 and 10; EC 50, half maximal effective concentration; GGOH, geranylgeraniol; HFD, high fat diet; LV-4, lumber vertebra 4; M-CSF, macrophage colony stimulating factor; MNC, mononuclear cells; NFATc1, nuclear factor of activated T-cells 1; OPG, osteoprotegerin; OPN, osteopontin; OSC, osteocalcin; OSX, osterix; PGE2, prostaglandin E2; RANKL, receptor activator of nuclear factor-κB ligand; RARβ, retinoic acid receptor beta; SMI, structural model index; Tb.N., trabecular number; Tb.Sp, trabecular separation; TGF-β1, -R1, 2, 3, transforming growth factor-beta 1, receptor 1, 2, 3; TNF, tumour necrosis factor; TRAP, tartrate-resistant acid phosphatase; VEGF 120, vascular endothelial growth factor 120; VEGFR2, vascular growth factor receptor 2; VSMCs, vascular smooth muscle cells; +/−, with or without; ↑, increase or upregulated; ↓, decrease or downregulated; ↔, no change.

## Data Availability

The original contributions presented in this study are included in the article and Supplementary Material. Further inquiries can be directed to the corresponding author.

## Supplementary Materials

The following supporting information can be downloaded at: https://www.mdpi.com/article/10.3390/ph19071117/s1, Table S1: search strategy; Table S2: list of excluded articles; Table S3: list of included articles.
